# Multi-visual pattern mining algorithm based on variational inference Gaussian mixture and pattern activation response map model

**DOI:** 10.1371/journal.pone.0334756

**Published:** 2025-11-11

**Authors:** Zhengyuan Zhang, Ping Chen, Yajun Liu, Yi He

**Affiliations:** College of Art and Design, Guangdong University of Science and Technology, Dongguan, China; Shandong Agricultural University, CHINA

## Abstract

Multi-visual pattern mining plays an important role in image classification, retrieval, and other fields. A multi visual pattern mining algorithm based on variational inference Gaussian mixture model and pattern activation response graph is introduced to address the issues of insufficient frequency and discriminability faced by traditional algorithms. The innovation of this algorithm lies in combining variational inference Gaussian mixture model with pattern activation response graph. The former solves the limitation of manually presetting the number of modes in traditional methods by determining the optimal number of modes to ensure frequency. The latter improves discriminability by capturing key areas of the image, solving the problem of traditional algorithms being difficult to balance the two and distinguish multiple patterns within the same category. The results showed that in quantitative analysis, the algorithm had a high frequency of 92.81% when the similarity threshold was 0.866 on the Canadian Institute for Advanced Research-10 dataset. On the Travel dataset, the classification accuracy and F1 value were as high as 95.36% and 94.17%, respectively, which were significantly higher than other algorithms. The proposed multi-visual pattern mining algorithm has high frequency and discriminability, which can provide a more comprehensive visual representation and help better mine images of the same category but different visual patterns. This algorithm provides technical support for image classification and retrieval.

## 1. Introduction

With the rapid development of computer vision technology, multi-visual pattern mining has become a core problem in the field of computer vision [1,2]. Multi visual pattern mining refers to automatically extracting a set of visual regions with dual characteristics from image data. One is frequency, that is, the pattern repeatedly appears in multiple images of the same category. The second is discriminability, which can not only distinguish different categories, but also identify different sub patterns formed within the same category due to differences in shooting angle, object posture, and local features. In tasks such as fine-grained image classification and cross perspective image retrieval, a single visual mode cannot cover the diverse visual representations of similar objects, while multi visual mode mining can improve the model’s ability to parse complex visual information by capturing fine-grained differences within categories. This technology is to extract visual regions with frequency and discriminability from images. This pattern is beneficial for in-depth recognition and understanding of image information, which plays an important role in fields such as image classification and retrieval [3,4]. In traditional visual pattern mining methods, frequency and discriminability are two core characteristics. Frequency refers to the frequent occurrence of patterns in multiple images of the same category, reflecting the representativeness of the patterns. Discriminability requires patterns to be able to distinguish visual differences between different categories. Normally, an effective visual pattern should have good discrimination between different categories and frequently appear in different images of the same category [5,6]. Traditional visual pattern mining methods only focus on one attribute. In terms of discriminability, traditional methods mainly use deep learning algorithms to select representative features and improve their discriminability ability, but they do not consider the frequency of patterns, resulting in an inability to reflect the overall features of the image well. In terms of frequency, traditional methods focus on extracting patterns that appear in most images, but do not consider the discriminative nature of patterns, making it difficult to effectively distinguish images [7]. At present, there are methods that comprehensively consider two characteristics, but most methods assume that patterns of the same category are single, making it difficult to distinguish different visual patterns of the same category [8]. Therefore, how to identify and distinguish these different visual patterns within the same category has become a major challenge in current research on visual pattern mining.

In the field of computer vision, mining key information in images is crucial [9]. To improve the model’s ability to learn subtle differences and fine-grained information, Z. Shen et al. proposed an unsupervised image blending method to address the over-fitting problem of Siamese frameworks in unsupervised learning. The results showed that the unsupervised learning model performed well in image representation learning on datasets such as CIFAR-10 and CIFAR-100, and had significant advantages in capturing detailed information and over-fitting [10]. M. H. Guo et al. proposed an attention-based method for region recognition in complex scenes in computer vision, which mimicked the human visual system for dynamic weight adjustment. The results indicated that the attention mechanism could effectively improve task performance in image visual pattern mining, and performed well in high-dimensional image features and multi-modal learning [11]. Y. Bi et al. proposed a comprehensive evolutionary computation method framework to address the high dimensionality, distortion, and complexity in image analysis tasks. This framework included key tasks such as edge detection, image segmentation, feature analysis, image classification, and object detection. The results indicated that the evolutionary computation method had high feature extraction and classification accuracy when dealing with complex image problems [12]. Z. Wang et al. analyzed the progress of Generative Adversarial Networks (GANs) in high-quality image generation, diversity enhancement, and training stabilization in computer vision. The results showed that the currently popular GAN architecture achieved significant results in generating trustworthy images, image to image translation, and other fields [13]. T. M. Ward et al. proposed a computer vision method based on deep neural networks for image understanding and discrimination in intraoperative video analysis. This method utilized deep learning models to accurately identify key steps and instruments during the surgical process. The results showed that deep neural networks were significantly superior to traditional machine learning methods in terms of accuracy and real-time performance in image recognition [14]. In response to the problem of traditional methods being cumbersome and inefficient in fish image classification, R. M. Aziz et al. proposed an optimization model based on deep learning artificial neural networks. This model combined cuckoo search algorithm with genetic algorithm, aiming to improve the visual pattern mining effect of fish images. Compared with other deep learning techniques, the proposed optimization method outperformed in evaluation metrics such as classification accuracy, recall rate, and F1 value [15].

Variational reasoning technique is a powerful Bayesian inference method widely used in complex data modeling and inference tasks. N. Manouchehri et al. proposed a finite mixture model based on multivariate beta distribution for the generation and information extraction of large-scale complex data. Simultaneously, the variational inference technique was used to solve parameter estimation problems. The experimental results showed that this method had excellent performance in medical applications such as multi-class colon tissue analysis and colorectal cancer image segmentation [16]. To address the data distribution shift caused by time-varying behavior in industrial processes, Q. Dai et al. proposed an incremental variational Bayesian Gaussian mixture model. This method adapted to changes in data distribution through a decreasing optimized Gaussian mixture model. Simultaneously, the physical interpretation was taken to construct monitoring schemes, achieving differentiation between faults and normal offsets. The results indicated that this method could accurately identify faults and effectively respond to time-varying dynamics [17]. Y. Su et al. proposed a new unsupervised model that combined Gaussian mixture variational auto-encoder and one-dimensional convolutional neural network to detect abnormal machine instances in response to multiple categories and lack of labels faced by multivariate time series in anomaly machine instance detection. The experiment showed that the area under the receiver operation characteristic curve was 0.99, and the F1 value was as high as 0.94 [18]. L. Xie et al. proposed a ship trajectory data anomaly detection model based on Gaussian mixture variational auto-encoder to address the limitations of learning ability in multi-class ship trajectory anomaly detection. The results showed that the model achieved a detection rate of 91.26% and a false positive rate of 0.68% on the coastal dataset in the United States, outperforming traditional models [19]. To solve the frequent occlusion and dense pedestrian detection, H. He et al. proposed a variational line pedestrian detector, which improved the positioning accuracy by formulating pedestrian detection as a variational inference problem. This method estimated the posterior probability through a probability model and used reparameterization techniques to improve detection accuracy in highly occluded scenes. The results showed that this method outperformed traditional methods on datasets such as CrowdHuman and CityPersons [20]. T. Zhang et al. proposed a variational Bayesian algorithm to address the limitations of Gaussian models in processing datasets containing non-Gaussian features. It was used for fast and scalable inference of potential non-Gaussian models. The results showed that the algorithm effectively reduced the impact of extreme events in the potential process and improved the inference robustness [21].

In summary, domestic and foreign researchers have conducted extensive research on image information mining and classification in the field of computer vision, and have made significant progress in the combination of unsupervised learning, pattern recognition, and deep learning technologies. However, these methods have certain limitations in mining complex scenarios and fine-grained patterns. Among them, the specific types of complex scenes include multi-modal feature distributions of the same type, fine-grained visual differences, and high-dimensional feature noise interference. The multimodal feature distribution of the same type refers to the multimodal feature distribution formed by the differences in shooting angles, local features of objects, and pose changes in the same type of images. Fine grained visual differences refer to subtle but crucial distinguishing features within a category. High dimensional feature noise interference refers to the high dimensionality and redundant information of image features, resulting in effective patterns being masked by noise. In the process of image classification and pattern mining, existing methods often rely on relatively simple feature extraction and fail to fully capture the diverse visual patterns within categories. To address this issue, a multi-visual pattern mining algorithm based on Variational Inference Gaussian Mixture-Pattern Activation Response Map (VIGMM-PARM) is proposed to achieve good visual pattern mining results. The innovation of the research lies in combining variational inference techniques and PARM, while considering the frequency and discriminability of patterns. VIGMM can adaptively determine the optimal number of patterns to ensure the frequency of multi-visual pattern mining. PARM is used to capture key regions in images, helping to mine and distinguish multiple visual patterns within the same category. Through this innovative combination, VIGMM-PARM performs better in complex scenarios, where VIGMM can adaptively model multimodal distributions of the same type and filter high-dimensional noise, and PARM can enhance fine-grained differences through independent feature representation. The two work together to adapt to multimodal features, enhance discriminative ability, and focus on effective modes. In addition, the limitation of traditional algorithms in terms of frequency is the need to preset the number of frequently occurring patterns in similar images, which can easily lead to inaccurate estimation of the number of patterns and result in insufficient frequency. In terms of distinguishability, it is difficult to effectively capture the unique semantics of key regions and distinguish different patterns within the same category. The VIGMM-PARM algorithm breaks through the limitations of traditional algorithms in terms of frequency and distinguishability through a dual mechanism. On the one hand, VIGMM is a Gaussian mixture model based on variational inference, which uses the Dirichlet process as a prior to automatically learn the number of frequently occurring patterns in similar images, avoiding the insufficient frequency caused by the preset number in traditional methods. On the other hand, PARM constructs independent activation response maps for each pattern, capturing the unique semantics of key regions through linear combination feature maps, effectively distinguishing different patterns within the same class, and solving the problem of insufficient discriminability.

The study reviewed previous research, with the first group introducing existing pattern mining methods in computer vision, and the second group introducing existing variational inference modeling techniques.

## 2. Methods and materials

To improve the frequency and discriminability of visual pattern mining, a multi-visual pattern Sampling (SES) strategy is introduced to optimize positive and negative samples and reduce the proportion of false positives. Simultaneously, PARM is used to represent pattern features. In response to SES’s inability to adaptively determine the optimal number of patterns, SES is further optimized. VIGMM is introduced to propose a multi-visual pattern mining algorithm based on VIGMM-PARM.

### 2.1. Construction of ses-parm mining algorithm

**Fig 1:**
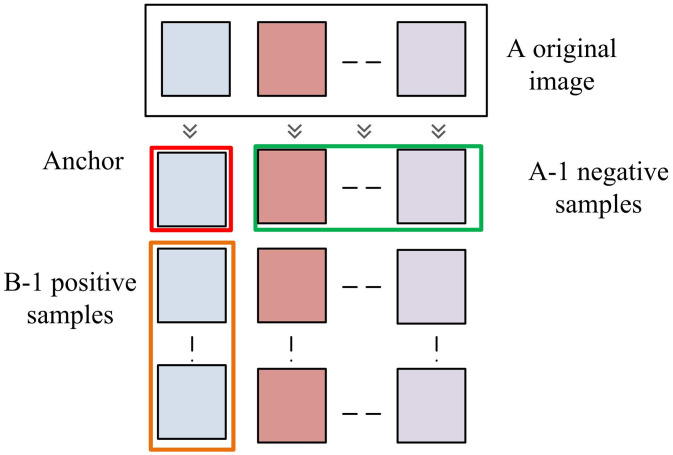
Self-enhancement strategy flowchart.

In multi-visual pattern, both labels and quantities are unknown. Multi visual pattern mining is the process of extracting a set of visual regions from an image that possess both frequency and discriminative properties. It can capture fine-grained differences within categories, providing more comprehensive visual information support for tasks such as fine-grained classification and cross perspective retrieval. In practical applications, images contain multiple potential visual patterns, which originate from different shooting angles, different parts of objects, or changes in appearance [22]. In traditional contrastive learning, sampling strategies typically select images of the same category as positive samples and images of other categories as negative samples. After experiencing contrastive learning, images of the same category are made closer in the feature space, while images of different categories are made farther apart. However, due to the fact that all images of the same category are forced to cluster together, their feature space becomes “crowded” and cannot effectively distinguish different visual patterns [23,24]. Therefore, the study introduces SES strategy to select positive samples, as shown in Fig 1.

In Fig 1, in each training batch, there are A original images, and each image generates B enhanced versions. An anchor sample is selected in B, and the remaining B−1 samples are all positive samples. The enhanced versions of A−1 original images form negative samples. The relationship between the number of positive and negative samples is shown in equation (1).


$$B-1>\frac{A-1}{D}$$ (1)

**Fig 2:**
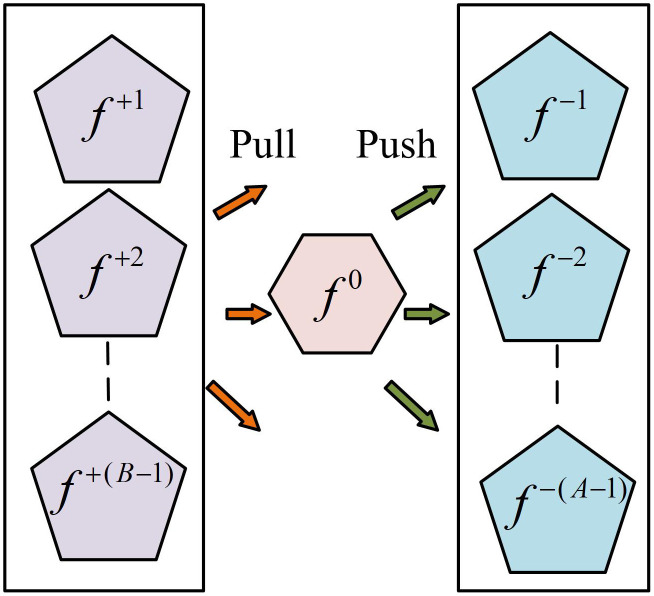
Operation diagram of self-enhancement sampling strategy.

In equation (1), D is the number of categories. This sampling method can effectively reduce the proportion of false positives and ensure that images with the same pattern features are closer. The operation diagram of SES strategy is shown in Fig 2.

In Fig 2, $f^{\;\;0}$ represents the anchor point sample. $f^{+}$ represents the feature vector of the positive sample. $f^{-}$ represents the feature vector of negative samples. “pull” refers to the operation of pulling the feature vector of the positive sample closer to the anchor sample $f^{\;\;0}$ to enhance its similarity in the feature space. “push” refers to the operation of pushing the feature vector of negative samples away from anchor sample $f^{\;\;0}$ to increase their distance in the feature space. To cooperate with the SES strategy, a loss function for metric learning is also required. The study mainly uses the Multi-Pattern (MP) loss function, whose expression is shown in equation (2).


$$Loss_{MP}=\frac{\text{1}}{B-1}\sum_{b=1}^{B-1}\;\log\frac{\exp(\sin(f^{0},f^{+b}))}{\sum_{b=1}^{B-1}\;\exp(\sin(f^{0},f^{+a}))+\sum_{a=1}^{A}\;\exp(\sin(f^{0},f^{-a}))}$$ (2)

**Fig 3:**
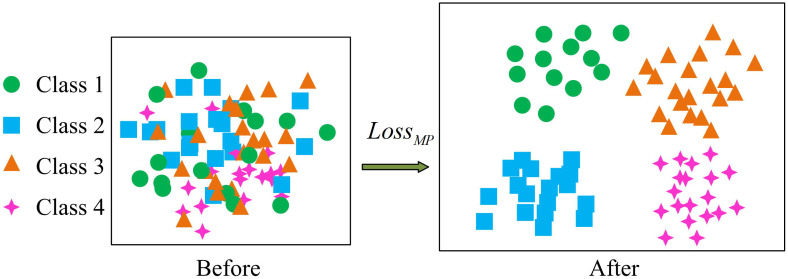
Schematic diagram of multi-pattern loss module.

In equation (2), $Loss_{MP}$ represents the definition of the MP loss function. $f^{-a}$ represents the feature vector of the a -th negative sample. $f^{+b}$ represents the feature vector of the b -th positive sample. The schematic diagram of the MP loss module is shown in Fig 3.

**Fig 4:**
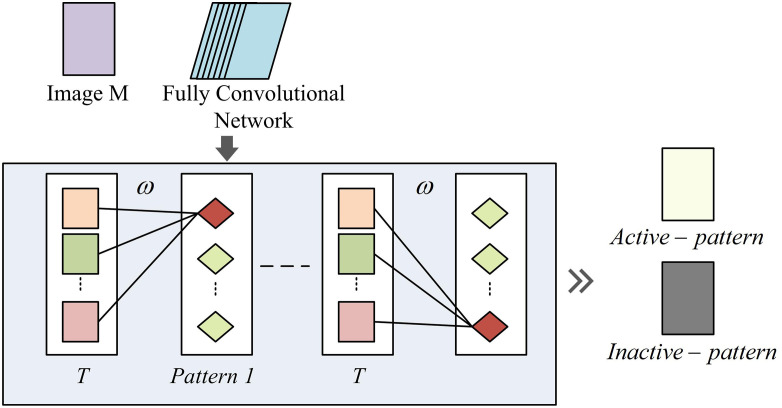
Schematic diagram of PARM.

In Fig 3, when the MP loss function is not used, the features of different patterns will be confused together. However, after applying the MP loss function, the features of the same pattern will be combined together, and there will be a large gap between different patterns, which improves the frequency of the model. To effectively represent the feature space of different patterns, a feature representation method based on linear combination, namely PARM, is further proposed. This method sets linear combinations for each pattern category, and each linear combination represents the network’s activation of the corresponding pattern. Through this method, different patterns have independent feature representations, and there will be no crowding of pattern features. The schematic diagram of PARM is shown in Fig 4.

In Fig 4, given an image M, it is input into a fully convolutional network. T represents the feature map extracted by the last convolutional layer, and the number of feature map channels is C. These feature maps are multiplied with different weights ω to generate different patterns pattern. The final activated pattern active−pattern and inactive pattern inactive−pattern are generated through linear combination. This method can expand a single combination into E combinations, where each combination generates a pattern activation response map. Among them, E represents the maximum number of patterns. The activation mapping expression for the e -th pattern in the d -th category in image M is shown in equation (3).


$$P^{d,e}=\sum_{c}\omega_{c}^{d,e}T_{c}$$ (3)

In equation (3), $P^{d,e}$ represents the activation status of the e -th pattern in the d -th category. $\omega_{c}^{d,e}$ represents the weight of the feature map, and the larger its value, the more semantic information the feature map contains [25]. The global maximum value of $P^{d,e}$ can characterize the strength of discriminability, as shown in equation (4).


$$Z^{d,e}=\max pool(P^{d,e})$$ (4)

In equation (4), $Z^{d,e}$ represents the global maximum value of the e -th pattern in the d -th category. The vector formed by the global maximum values of all patterns is the feature encoding of image M. The study uses the maximum global maximum value in category d as the score for that category, as shown in equation (5).


$$S_{d}=\max\left\{d^{e},e=1,2,\ldots ,E\right\}$$ (5)

In equation (5), $S_{d}$ represents the score of image M in each category. After softmax processing, the probability of category d will be obtained, as shown in equation (6).


$$\hat{y_{d}}=\frac{\exp(S_{d})}{\sum_{d}\;\exp(S_{d})}(d=1,2,...,D)$$ (6)

To minimize the difference between $\hat{y_{d}}$ and label $y_{d}$ and extract visual patterns with stronger discriminability, the study takes a cross entropy loss function for processing, which is expressed as equation (7).


$$Loss_{CE}=\sum_{d=1}^{D}\;-y_{d}\log({\hat{y}}_{d})$$ (7)

In equation (7), $Loss_{CE}$ represents the cross entropy loss function. By combining the MP loss function, the total loss function is shown in equation (8).


$$L=\lambda \cdot Loss_{CE}+(1-\lambda )\cdot Loss_{MP}$$ (8)

In equation (8), λ represents the balance weight. By combining SES and PARM, a multi-visual pattern mining algorithm based on SES-PARM is obtained, which balances frequency and discriminability in multi-visual pattern mining of the same category.

### 2.2. Multi-visual pattern mining based on vigmm-parm

**Fig 5:**
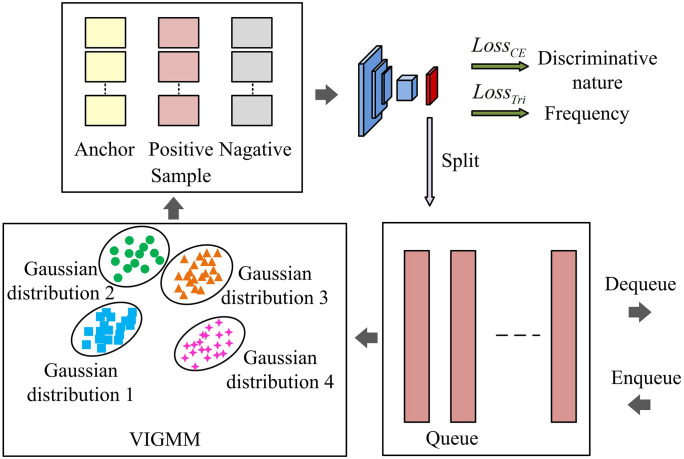
Framework diagram of multi-visual pattern mining algorithm based on VIGMM.

The SES-PARM-based multi-visual pattern mining algorithm mainly improves mining performance by setting multiple patterns for the same category. However, the number of categories for certain visual patterns is extremely small, and the SES-PARM method requires specifying the number of patterns in advance when processing such patterns, which poses a computational redundancy problem and affects frequency [26,27]. Therefore, based on the original SES, the VIGMM model is further proposed to automatically determine the optimal number of patterns and ensure the frequency of multi-visual pattern mining. This mainly uses feature queues to increase the number of negative samples and achieve better metric learning. The framework diagram of the multi-visual pattern mining algorithm based on VIGMM is shown in Fig 5.

In Fig 5, the multi-visual pattern mining algorithm based on VIGMM mainly treats each visual pattern as a Gaussian distribution, and maintains a feature queue for each category to store the historical features of that category. The feature queue supports Enqueue and Dequeue operations. The sample types include anchor points, positive samples, and negative samples. The frequency loss function of the pattern is a triplet loss function, while the discriminative loss function of the pattern is a cross entropy loss function. VIGMM can adaptively determine the number of patterns without the need to specify the number of patterns in advance. Through iterative optimization, it ensures that the mined visual patterns have both frequency and discriminability. In VIGMM, the GMM model is a generative model that assumes that the data is a mixture of multiple Gaussian distributions. Each data point is considered to be generated from a Gaussian distribution. In multi-visual patterns, assuming that each pattern follows a Gaussian distribution, the distribution expression for category d is shown in equation (9).


$$p(x^{d})=\sum_{k=1}^{K}\pi_{k}^{d}N(x^{d}\;|\;\mu_{k}^{d},\Sigma_{k}^{d})$$ (9)

In equation (9), $x^{d}$ represents the feature vector of category d. $\pi_{k}^{d}$ represents the probability density of the k -th component. $\mu_{k}^{d}$ represents the mean vector. $\Sigma_{k}^{d}$ represents the covariance matrix. Each Gaussian distribution has two main parameters, including covariance matrix and mean vector. The mean vector can be seen as a representative of visual patterns, which is the most representative sample position for this category. GMM assumes that the data comes from a mixture of multiple Gaussian distributions, with each data point having an implicit class label. In this method, the Dirichlet process is used as a prior to generate infinite Gaussian components through random probability measures, enabling the model to handle unknown numbers of patterns. In the modeling process, the conditional distribution of the feature queue is constructed on the mean and covariance matrix of latent variables and Gaussian components, further relying on the random probability measures generated by the Dirichlet process. Meanwhile, based on the Bayesian inference method, the posterior distribution of the latent variable is calculated. However, when solving GMM, the number of visual patterns contained in each category is uncertain. Therefore, the study adopts variational inference method to approximate the posterior distribution of GMM. In the variational inference framework, the goal is to find a simple variational distribution to approximate the complex true posterior distribution. According to the basic principle of variational inference, approximation is achieved by minimizing the KL divergence between the variational distribution and the true posterior distribution. In order to approximate the posterior distribution of GMM, the relevant information of the feature queue is integrated into the joint distribution. Finally, through the core deduction logic of variational inference mentioned above, the variational lower bound expression for approximating the posterior distribution is obtained. According to information theory, approximation is achieved by minimizing the KL divergence between the two, as shown in equation (10).


$$KL(\tau (\Theta )\|p(\Theta |Q))=\int \tau (\Theta )\ln\frac{\tau (\Theta )}{p(\Theta |Q)}d\Theta \to \min$$ (10)

Where, τ(Θ) is a simple variational distribution. p(Q,Θ) is a complex true posterior distribution. Θ represents the latent variable. Q represents the feature queue. By Bayes’ theorem, we get $p(\Theta |Q)=\frac{p(Q,\Theta )}{p(Q)}$, which is substituted into the KL divergence and rearranged to obtain Equation (11).


KL(τ(Θ)‖p(Θ|Q))=∫τ(Θ)lnτ(Θ)dΘ−∫τ(Θ)lnp(Q,Θ)dΘ+lnp(Q) (11)

Since it is independent lnp(Q,Θ) of the variational distribution τ(Θ), minimizing the KL divergence is equivalent to maximizing the variational lower bound L(τ). The specific expression is shown in Equation (12).


L(τ)=∫τ(Θ)lnp(Q,Θ)dΘ−∫τ(Θ)lnτ(Θ)dΘ (12)

Further L(τ) simplification, using logarithmic properties $\ln p(Q,\Theta )=\ln\frac{p(Q,\Theta )}{\tau (\Theta )}\cdot \tau (\Theta )$ and combining integral expansion, can be sorted out to obtain equation (13).


$$L(\tau )=\int \tau (\Theta )ln(\frac{p(Q,\Theta )}{\tau (\Theta )})d\Theta$$ (13)

**Fig 6:**
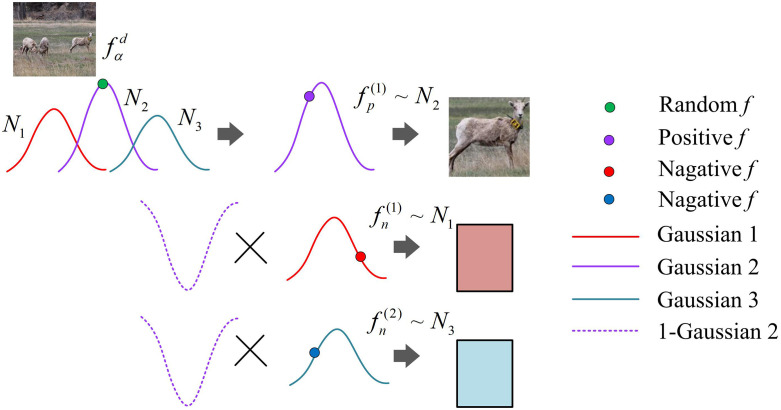
Sampling diagram of VIGMM (Source from: https://www.usgs.gov/media/images/bighorn-sheep-montana).

In terms of frequency, VIGMM not only considers self-enhancement samples, but also constructs a feature opposition model through variational inference method, and selects the optimal samples to calculate the metric loss value. The steps for VIGMM to automatically determine the optimal number of modes are to first introduce Dirichlet process as the prior distribution of Gaussian components, allowing the model to generate theoretically infinite potential modes. Then use variational inference to optimize the variational lower bound and approximate the posterior distribution to estimate the weights of each Gaussian component. In iterative optimization, Gaussian components with weights below the threshold are automatically removed, and only the components with statistical significance are retained, which is the optimal number of patterns. This process does not require manual preset and achieves adaptive adjustment of the number of modes. The sampling diagram of VIGMM is shown in Fig 6.

In Fig 6, $f_{\alpha }^{d}$ represents the anchor point of category d. Gaussian1, Gaussian2, and Gaussian3 represents the three optimal Gaussian distributions, each defined by its mean and covariance matrix. Positivef represents positive samples. Negativef represents negative samples. Randomf represents random samples. $f_{p}^{\left(1\right)}$ represents a positive sample. $f_{n}^{\left(1\right)}$ and $f_{n}^{\left(2\right)}$ represent two negative samples. The anchor point is located in Gaussian distribution $k^{\prime }$, and its expression is shown in equation (14).


$$k^{\prime }=\mathrm{\backslash arg}\underset{k}{\max}\;N(f_{\alpha }^{d}\;|\;\mu_{k},\Sigma_{k})$$ (14)

After determining the Gaussian distribution of the anchor points, effective positive samples can be selected. Through metric learning, anchor points can maintain greater differences from negative sample featusres and smaller differences from positive sample features. In addition, to ensure a greater difference between negative samples and anchor points, a sampling strategy for negative samples is established, as expressed in equation (15).


$$p(f_{n}^{d})\sim \sum_{k=1;k\neq k^{\prime }}^{K}\;s_{k}\frac{N(x\;|\;\mu_{k},\Sigma_{k^{\prime }})(1-N(x\;|\;\mu_{k^{\prime }},\Sigma_{k^{\prime }})}{\int_{-\infty }^{+\infty }\;N(x\;|\;\mu_{k},\Sigma_{k^{\prime }})(1-N(x\;|\;\mu_{k^{\prime }},\Sigma_{k^{\prime }})dx}$$ (15)

In equation (15), $s_{k}$ is the one hot encoding with a value of 0–1, used to estimate the number of visual patterns in the image. To improve frequency, the distance between anchor points and each sample is mainly calculated using a ternary loss function, as shown in equation (16).


$$Loss_{Tri}=\sum_{i}^{N}\;[∥f_{\alpha }^{d}-f_{p}^{d}{∥}_{2}^{2}-∥f_{\alpha }^{d}-f_{p}^{d}{∥}_{2}^{2}+\beta {]}_{+}$$ (16)

In equation (16), β represents the minimum interval parameter. To ensure discriminability, the cross entropy loss function is also used for optimization, and the overall loss function is shown in equation (17).


$$L=\lambda \cdot Loss_{Tri}+Loss_{CE}$$ (17)

**Fig 7:**
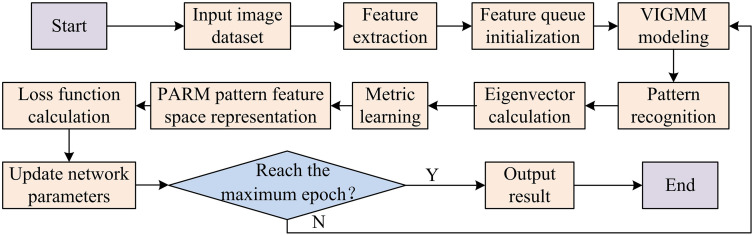
Flowchart of multi-visual pattern mining based on VIGMM-PARM.

In equation (17), the value of λ is 0.8. The flowchart of multi-visual pattern mining based on VIGMM-PARM is shown in Fig 7.

As shown in Fig 7, the multi-visual pattern mining process based on VIGMM-PARM first initializes the network parameters and creates an empty feature queue for each category. Then, the VIGMM is used to model the extracted features and model the visual pattern as a Gaussian distribution. The appropriate visual pattern is selected through VIGMM and adaptively determined the number of patterns. Next, the feature vector is calculated. The frequency of optimization patterns is measured to select appropriate sample pairs for optimization. Subsequently, PARM is used to independently represent the feature spaces of different patterns, avoiding feature crowding. The total loss function is calculated again, including metric learning loss (ternary loss function) and classification task loss (cross entropy loss function). Whether the preset epoch has been reached is determined. If not, the feature queue is updated and the visual pattern is re-determined. If achieved, the mined multi-visual pattern features, as well as the number of patterns and corresponding feature representations for each category are output. The multi-visual pattern mining algorithm based on VIGMM-PARM mainly models visual patterns as Gaussian distributions and models them through GMM. Meanwhile, the variational inference method is used to estimate the distribution range of patterns and adaptively determine the number of patterns. The frequency and discriminability of patterns are optimized through metric learning and classification tasks, respectively. By selecting appropriate samples for optimization, adaptive multi-visual pattern mining is achieved after multiple iterations, which improves frequency while ensuring discriminative performance.

## 3. Results

To verify the effectiveness of the VIGMM-PARM-based multi-visual pattern mining algorithm, qualitative and quantitative analyses are conducted to evaluate the frequency and discriminability of different algorithms. In quantitative analysis, the experiment first conducts ablation experiments to verify the superiority of each module in VIGMM-PARM. Next, the VIGMM-PARM algorithm is compared with other algorithms to highlight the advantages of multi-visual pattern mining algorithms based on VIGMM-PARM. In qualitative analysis, the visual representation effects of different algorithms are compared.

### 3.1. Quantitative analysis

To evaluate the effectiveness of the VIGMM-PARM-based multi-visual pattern mining algorithm, the quantitative analysis is conducted to assess the frequency and discriminability of different algorithms. The discriminability evaluation indicators include classification accuracy and F1 value. The frequency evaluation method is FR, which is used to quantify the similarity of visual representations discovered in the high-level feature space. The proportion of similar images in the dataset is calculated to measure the frequency of a certain visual representation. Three similarity thresholds are set in the validation, namely 0.866, 0.892, and 0.940. The basic network model used in the study is ResNet-50, which freezes the pre training parameters of the first 10 layers and selects the second to last convolutional layer output as the feature extraction layer, with an output feature dimension of 2048. The weight dimension of the linear combination in the PARM module matches the number of channels in the feature map, set to 512. The optimizer uses Adam, with an initial learning rate of 0.001, and the learning rate decays to 0.5 every 20 epochs. The batch size is fixed at 32, and the total number of training epochs is 100. The concentration parameter of the Dirichlet process in VIGMM is set to 0.1, and the initial search range for Gaussian components is 3–15. The experiment mainly compares the frequency of various algorithms at different thresholds. The experiment is conducted under the PyTorch framework, using a computer system with NVIDIA 2080 Ti graphics card and 10GB of memory. The experimental dataset includes the Canadian Institute for Advanced Research-10 (CIFAR-10) and the Travel dataset. The CIFAR-10 dataset contains 10 categories of image datasets, each with 6,000 32 × 32 color images. The Travel dataset comes from the travel website TripAdvisor, where photos of each tourist attraction are considered as belonging to the same category. The detailed information of each dataset is shown in Table 1.

**Table 1:** Detailed information of each dataset.

Dataset	CIFAR-10	Travel
Number of categories	10	20
Training set	40,028	68,239
Validation set	9,863	17,486
Test set	9,826	18,693

**Fig 8:**
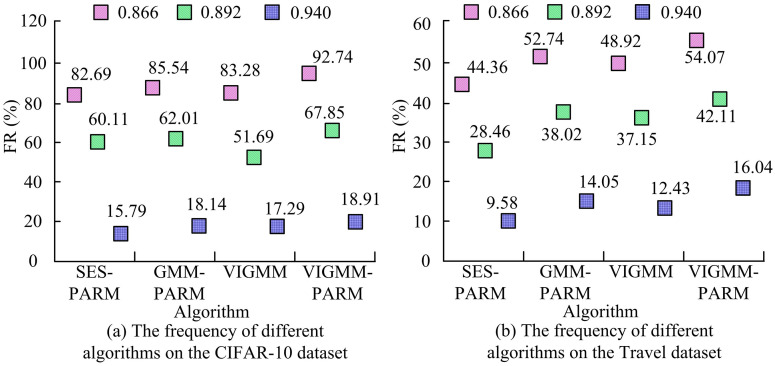
Frequency analysis of ablation experiments.

To verify the superiority of each part of the VIGMM-PARM algorithm, ablation experiments are conducted. The comparison methods used include SES-PARM, GMM-PARM, and VIGMM. The study compares the frequency of multi-visual pattern mining algorithms based on different methods on various datasets, and the results are shown in Fig 8. According to Fig 8(a), in the CIFAR-10 training set, when the similarity threshold was 0.866, the FR of the VIGMM-PARM algorithm was 92.74, while SES-PARM, GMM-PARM, and VIGMM were only 82.69%, 85.54%, and 83.28%, respectively. When the similarity threshold was 0.892, the FR of VIGMM-PARM algorithm was 67.85, while VIGMM was only 51.69%. When the similarity threshold was 0.940, the FR of VIGMM-PARM algorithm was 18.91, which was still higher than other algorithms. According to Fig 8(b), on the Travel dataset, the FR of the VIGMM-PARM algorithm was higher than that of other algorithms, reaching 54.07%, 42.11%, and 16.04% respectively at thresholds of 0.866, 0.892, and 0.940. The FR of SES-PARM algorithm at various thresholds was only 44.36%, 28.46%, and 9.58%, respectively, with significantly lower frequency than VIGMM-PARM algorithm. Meanwhile, the FR of GMM-PARM algorithm at various thresholds were only 52.74%, 38.02%, and 14.05%, respectively, all lower than that of VIGMM-PARM algorithm. VIGMM has more advantages compared with SES and GMM. In addition, the FR of the VIGMM algorithm at various thresholds were only 48.92%, 37.15%, and 12.43%, respectively, indicating that PARM could also affect the frequency of mining algorithms. Compared with other algorithms, VIGMM-PARM exhibited high frequency across different categories of datasets and similarity thresholds.

**Fig 9:**
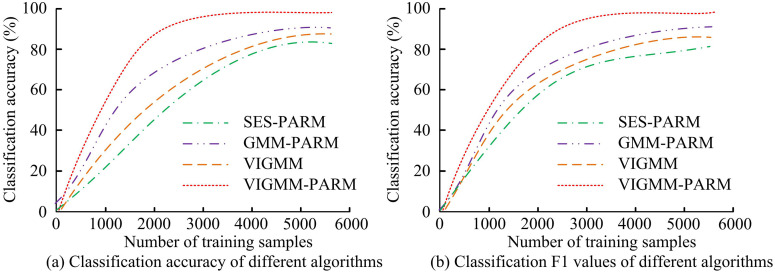
Discriminability analysis of ablation experiment.

The study then compares the discriminative performance of different methods on the CIFAR-10 training set, selecting 6,000 samples for training. The results are shown in Fig 9. As shown in Fig 9(a), the classification accuracy of VIGMM-PARM algorithm in the CIFAR-10 training set was higher than that of other algorithms, and the training speed was faster. When the number of training samples reached around 3,000, the classification accuracy of the VIGMM-PARM algorithm stabilized at 95.82%. The SES-PARM algorithm only tended to converge when the number of training samples reached around 5000, and the classification accuracy was only 80.61%. The classification accuracy of GMM-PARM algorithm and VIGMM algorithm after training was only 90.04% and 83.12%, respectively. As shown in Fig 9(b), the F1 value of the VIGMM-PARM algorithm during the training phase was 94.25%, while SES-PARM, GMM-PARM, and VIGMM were only 78.24%, 87.21%, and 81.69%, respectively, all lower than the VIGMM-PARM algorithm. This confirms the superiority of VIGMM and PARM in multi-visual pattern mining.

**Fig 10:**
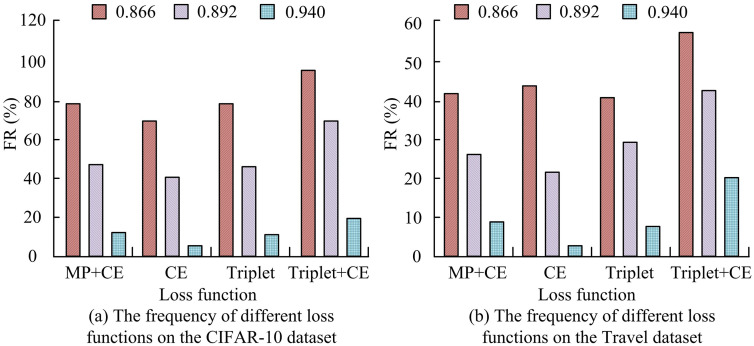
The frequency of different loss functions on various datasets.

To verify the effectiveness of the loss function in the VIGMM-PARM algorithm, different loss functions are compared. The loss function used in the research algorithm is Triplet+CE, and the comparison loss functions are CE, Triplet, and MP + CE, respectively. The frequency of mining algorithms using different loss functions on different datasets is shown in Fig 10. As shown in Fig 10(a), on the CIFAR-10 dataset, when using Triplet+CE as the loss function in the VIGMM-PARM algorithm, the FR was the highest. At similarity thresholds of 0.866, 0.892, and 0.940, the FR of the algorithm using Triplet+CE loss function reached 95.46%, 70.11%, and 20.24%, respectively. When using CE as the loss function, the FR of the algorithm at each threshold was only 70.12%, 39.68%, and 5.01%, respectively. The Triplet loss function could effectively improve the frequency of the algorithm. Meanwhile, when only Triplet was used as the loss function, the FR of the algorithm at various thresholds were only 79.71%, 43.52%, and 9.58%, respectively, indicating that the CE loss function could also improve the frequency of the algorithm. In addition, when using MP + CE as the loss function, the FR of the algorithm at each threshold was 79.85%, 43.68%, and 11.01%, all lower than the Triplet+CE loss function. The Triplet loss function was superior to the MP loss function. From Fig 10(b), on the Travel dataset, when using Triplet+CE as the loss function, the FR of the algorithm at different thresholds were as high as 58.25%, 43.15%, and 19.17%, respectively, which was better than the other loss functions. Taking Triplet+CE as the loss function of VIGMM-PARM algorithm can effectively improve the frequency of the algorithm.

**Fig 11:**
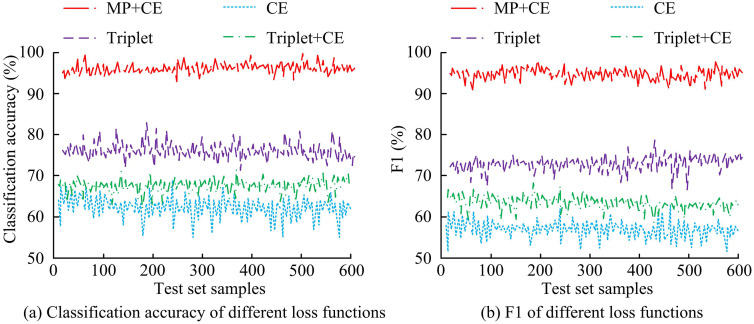
Discriminability of different loss functions on the Travel dataset.

The discriminative performance of mining algorithms with different loss functions on the Travel test set is tested by extracting 600 test samples, and the results are shown in Fig 11. As shown in Fig 11(a), on the Travel dataset, when Triplet+CE was used as the loss function in the VIGMM-PARM algorithm, the classification accuracy of the algorithm was the highest, with an average classification accuracy of 95.14% in the test samples. The average classification accuracy of CE, Triplet, and MP + CE loss functions were 61.68%, 68.42%, and 76.81%, respectively, which were significantly lower than the Triplet+CE loss function. According to Fig 11(b), the algorithm using Triplet+CE as the loss function had higher F1 value, at 94.82%. The F1 values of CE, Triplet, and MP + CE loss functions were only 56.91%, 65.37%, and 73.04%, respectively. The Triplet+CE loss function used in the study could effectively improve the discriminability.

To verify the superiority of the VIGMM-PARM-based multi-visual pattern mining algorithm, several advanced multi-visual pattern mining algorithms are further selected for comparison. The comparative algorithms include Mode-Deep Pattern Mining (MDPM), Piecewise Convolutional Neural Network (P-CNN), and Deep Neural Network Feature Density Clustering (DNN-FDC). The frequency and discriminability of each algorithm in different datasets are shown in Table 2. According to Table 2, the VIGMM-PARM algorithm outperformed other algorithms in terms of frequency and discriminative performance on various datasets. On the CIFAR-10 dataset, the VIGMM-PARM algorithm achieved a high FR of 92.81% at a similarity threshold of 0.866, while the MDPM, P-CNN, and DNN-FDC algorithms only achieved FR of 72.28%, 82.96%, and 87.25%, respectively. Meanwhile, the classification accuracy and F1 value of VIGMM-PARM algorithm were as high as 95.82% and 94.28%, respectively, which were higher than MDPM, P-CNN, and DNN-FDC algorithms. The classification accuracy of MDPM, P-CNN, and DNN-FDC algorithms was 69.41%, 84.76%, and 92.15%, respectively, with F1 values of 64.82%, 82.15%, and 90.04%, respectively. In addition, the VIGMM-PARM algorithm outperformed other algorithms in terms of FR, classification accuracy, and F1 value on the Travel dataset, demonstrating its superiority and stability on different datasets.

**Table 2:** The frequency and discriminability of various algorithms on different datasets.

Algorithm	Data set	Frequency FR (%)			Discriminative nature	
		0.866	0.892	0.940	Classification accuracy (%)	F1 value (%)
MDPM	CIFAR-10	72.28	31.04	10.07	69.41	64.82
	Travel	43.87	20.17	8.75	65.84	63.15
P-CNN	CIFAR-10	82.96	49.67	14.74	84.76	82.15
	Travel	48.13	37.48	13.84	82.47	80.91
DNN-FDC	CIFAR-10	87.25	52.82	18.11	92.15	90.04
	Travel	50.12	38.96	14.08	88.82	84.75
VIGMM-PARM	CIFAR-10	92.81	70.15	20.47	95.82	94.28
	Travel	56.92	43.71	17.02	95.36	94.17

To further verify the impact of different initialization strategies on the stability of pattern mining in VIGMM, three different initialization schemes were designed in the experiment. (1) Random initialization: The mean and covariance matrix of Gaussian distribution are randomly generated; (2) K-means initialization: Based on the K-means clustering results, the cluster centers are used as the initial mean of the Gaussian distribution; (3) Class center initialization: Select the features of each class center sample as the initial mean of the Gaussian distribution. On the CIFAR-10 and Travel datasets, experiments were repeated 5 times for each strategy, and the FR, classification accuracy, and F1 values were calculated for each strategy. The results are shown in Table 3. According to Table 3, among the three initialization strategies, K-means initialization has the highest mean values for all indicators on both datasets. On the CIFAR-10 dataset, the FR of K-means initialization strategy reached 92.74%, the classification accuracy was as high as 95.82%, and the F1 value was as high as 94.25%, which were 3.11%, 3.67%, and 3.17% higher than random initialization, respectively. On the Travel dataset, the FR value of the K-means initialization strategy is 56.92%, the classification accuracy is as high as 95.36%, and the F1 value is as high as 94.17%, which is superior to other strategies. This indicates that K-means initialization not only improves the performance of pattern mining, but also reduces parameter fluctuations by utilizing data distribution priors, effectively enhancing algorithm stability.

**Table 3:** FR, classification accuracy, and F1 score under various strategies.

Initialization Strategy	Dataset	FR (%)	Classification Accuracy (%)	F1 (%)
Random	CIFAR-10	89.63 ± 3.82	92.15 ± 2.56	91.08 ± 2.71
	Travel	52.37 ± 3.15	91.24 ± 2.18	90.05 ± 2.34
K-means	CIFAR-10	92.74 ± 1.24	95.82 ± 0.87	94.25 ± 0.93
	Travel	56.92 ± 1.03	95.36 ± 0.76	94.17 ± 0.82
Class-center	CIFAR-10	90.85 ± 2.31	93.57 ± 1.64	92.43 ± 1.75
	Travel	54.63 ± 1.98	93.18 ± 1.32	92.06 ± 1.46

### 3.2. Qualitative analysis

**Fig 12:**
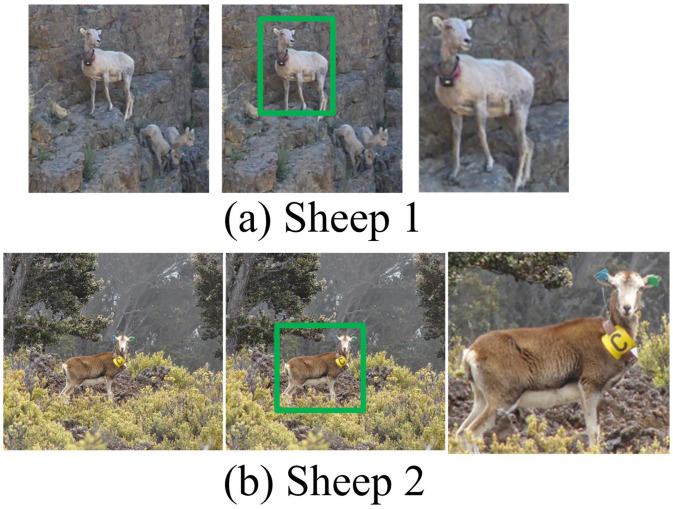
Visual representation of different categories by VIGMM-PARM (Source from: Picture (a): https://www.usgs.gov/media/images/highlands-bighorn-sheep-montana; Picture (b): https://www.usgs.gov/media/images/female-mouflon-sheep-stands-broadside).

To demonstrate the effectiveness of the VIGMM-PARM-based multi-visual pattern mining algorithm more intuitively, qualitative experiments are conducted. On the Travel datasets, the visual representation of different categories using this method is shown in Figure 12. In Fig 12, the first column of each image represents the original image, the second column represents the pattern activation response map, and the third column represents the mined visual representation. From Fig 12, the multi-visual pattern mining algorithm based on VIGMM-PARM could extract key information from images and effectively represent the corresponding categories. Meanwhile, this method could locate regions with strong discriminability within the same category. Among them, the multi visual pattern mining algorithm based on VIGMM-PARM can effectively distinguish the types of sheep by mining visual representations.

## 4. Discussion and conclusion

A multi-visual pattern mining algorithm based on VIGMM-PARM was proposed for the multi-visual pattern mining in the field of computer vision. The results showed that on the CIFAR-10 dataset, the frequency of the VIGMM-PARM algorithm reached 92.74% at a threshold of 0.866, far exceeding the SES-PARM, GMM-PARM, and VIGMM algorithms. On the Travel dataset, the frequency of VIGMM-PARM was consistently higher than other algorithms. As the threshold increased, FR remained at a high level. In the discriminative test of the CIFAR-10 dataset, the VIGMM-PARM algorithm had a classification accuracy of 95.82% and an F1 value of 94.25% during the training phase. The classification accuracy and F1 value of SES-PARM algorithm were only 80.61% and 78.24%, respectively. The classification accuracy and F1 value of GMM-PARM algorithm and VIGMM algorithm were lower than those of VIGMM-PARM algorithm. The reason is that VIGMM in the VIGMM-PARM algorithm can adaptively adjust the number of patterns through a Gaussian mixture model. This enables the algorithm to capture fine-grained visual differences in the high-dimensional feature space, thereby effectively partitioning and modeling multiple visual patterns. Compared with SES, VIGMM effectively improves the frequency of multi-visual pattern mining. Meanwhile, PARM can generate independent feature representations for each pattern through linear combination, avoiding the “crowding” problem of pattern features in traditional methods and enhancing the discriminability of patterns [28].

In the loss function comparison experiment, the VIGMM-PARM algorithm using Triplet+CE loss function performed the best in terms of frequency and discriminability on various datasets. On the CIFAR-10 dataset, when using the Triplet+CE loss function, the frequency reached a maximum of 95.46%. In contrast, the performance of the algorithm using CE or Triplet loss functions was significantly reduced. On the Travel dataset, when using the Triplet+CE loss function, the classification accuracy and F1 value were also superior to other loss functions. The reason is that Triplet loss ensures that the model can learn highly discriminative feature representations, while CE loss ensures that the activation responses of each category can be effectively classified [29].

The VIGMM-PARM algorithm showed significantly better frequency and discriminative performance on different datasets compared with MDPM, P-CNN, and DNN-FDC algorithms. On the CIFAR-10 dataset, the VIGMM-PARM algorithm achieved a FR of 92.81% at a similarity threshold of 0.866, while the MDPM, P-CNN, and DNN-FDC algorithms only achieved FR of 72.28%, 82.96%, and 87.25%, respectively. Meanwhile, the classification accuracy and F1 value of VIGMM-PARM algorithm were 95.82% and 94.28%, respectively, which were higher than MDPM, P-CNN, and DNN-FDC algorithms. Although MDPM adopts deep pattern mining methods, its fixed pattern construction method cannot fully adapt to the high-dimensional and complex pattern distribution in the data. In contrast, VIGMM-PARM can better capture visual differences in data by dynamically adjusting the pattern number and structure. Meanwhile, P-CNN mainly uses convolutional neural networks for pattern recognition, relying on predefined local patterns and lacking global similarity measures, which limits its performance in complex patterns [30]. DNN-FDC mainly utilizes deep neural networks for feature extraction and classifies samples through density clustering. However, this method relies on the feature density assumption, which is susceptible to noise or over-fitting issues in high-dimensional datasets and complex visual patterns. In addition, the Graph Regularized Discriminative Non negative Matrix Factorization (GDNMF) algorithm proposed by Z. Liu et al. provides a new approach for clustering and classification of visual data. This method addresses the limitations of traditional non negative matrix factorization and its variants that fail to fully utilize label information and local geometric structures. It achieves improvement by introducing two constraint terms in the objective function. The effectiveness of this method has also been confirmed through experiments on multiple public image datasets [31]. However, the core limitation of the GDNMF algorithm is that it does not take into account the challenges of multi view data processing, making it difficult to cope with complex scenes with multiple visual modes in the same category. In contrast, the proposed VIGMM-PARM algorithm not only adaptively captures multimodal distributions in similar data through VIGMM, but also constructs independent activation response maps for each pattern through PARM. While preserving local structural information, discriminative learning of multiple visual modes is more flexibly handled.

In summary, the VIGMM-PARM algorithm proposed in the study can effectively improve the frequency and discriminability of multi-visual pattern mining. However, this algorithm has certain limitations on real-time performance. Subsequent research will explore how to improve the computational efficiency of the VIGMM-PARM algorithm, so that it can quickly process and respond to real-time tasks.

## Supporting information

S1 File Minimal Data Set Definition. (DOC)
