# Supplementary material for: Multi-visual pattern mining algorithm based on variational inference Gaussian mixture and pattern activation response map model
Source: PLoS One. 2025 Nov 11;20(11):e0334756. doi: 10.1371/journal.pone.0334756 (PMC12604772; doi:10.1371/journal.pone.0334756)
Supplement: S1 File — (DOC) [file pone.0334756.s001.doc]

**The data in Figure 8**

| Dataset | Algorithm | FR (%) | | |
| --- | --- | --- | --- | --- |
|  |  | 0.866 | 0.892 | 0.940 |
| CIFAR-10 | SES-PARM | 82.69 | 60.11 | 15.79 |
|  | GMM-PARM | 85.54 | 62.01 | 18.14 |
|  | VIGMM | 83.28 | 51.69 | 17.29 |
|  | VIGMM-PARM | 92.74 | 67.85 | 18.91 |
| Travel | SES-PARM | 44.36 | 28.46 | 9.58 |
|  | GMM-PARM | 52.74 | 38.02 | 14.05 |
|  | VIGMM | 48.92 | 37.15 | 12.43 |
|  | VIGMM-PARM | 54.07 | 42.11 | 16.04 |

**The data in Figure** 9

| Algorithm | Number of training samples during convergence | Classification accuracy (%) | F1 (%) |
| --- | --- | --- | --- |
| SES-PARM | 5243 | 80.61 | 78.24 |
| GMM-PARM | 4817 | 90.04 | 87.21 |
| VIGMM | 4728 | 83.12 | 81.69 |
| VIGMM-PARM | 3158 | 95.82 | 94.25 |

**The data in Figure 1**0

| Dataset | Loss function | FR (%) | | |
| --- | --- | --- | --- | --- |
|  |  | 0.866 | 0.892 | 0.940 |
| CIFAR-10 | MP+CE | 79.85 | 43.68 | 11.01 |
|  | CE | 70.12 | 39.68 | 5.01 |
|  | Triplet | 79.71 | 43.52 | 9.58 |
|  | Triplet+CE | 95.46 | 70.11 | 20.24 |
| Travel | MP+CE | 42.15 | 27.64 | 9.71 |
|  | CE | 43.25 | 22.47 | 3.58 |
|  | Triplet | 41.76 | 29.34 | 9.07 |
|  | Triplet+CE | 58.25 | 43.15 | 19.17 |

**The data in Figure 11**

| Loss function | Classification accuracy (%) | F1 (%) |
| --- | --- | --- |
| MP+CE | 76.81 | 73.04 |
| CE | 61.68 | 56.91 |
| Triplet | 68.42 | 65.37 |
| Triplet+CE | 95.14 | 94.82 |
